# Acquiring a doctoral degree: was it worth it?

**DOI:** 10.2144/fsoa-2019-0142

**Published:** 2020-01-29

**Authors:** Olumide A Odeyemi

**Affiliations:** 1 Research Performance & Analysis, Research Division, University of Tasmania, Launceston, Tasmania, Australia

**Keywords:** doctoral degree, doctoral research, PhD, PhD admissions, PhD students, PhD supervisor, postgraduate education, research scientist

## Abstract

When I started my doctoral degree a couple of years ago at the University of Tasmania in Australia, my enthusiasm for starting a doctoral degree with a scholarship was very high; however, along the way, due to various challenges, I began to ask myself: is acquiring a doctoral degree worth it? In this article, I provide a detailed account of how I started and completed my doctoral study and highlight the inherent lessons I learned.

## The starting point

I started my Master of Science (MSc) on 21 December 2009 at the National University of Malaysia, 6 years after I began to passionately desire a doctoral degree for my career progression. I completed that master’s degree in 2013 and it was awarded on 26 April 2014. It took me a long time because I was working alongside my studies, as I was not funded.

Towards the end of 2013, I started collating the admission requirements for the a Doctor of Philosophy (PhD) degree as stated by different institutions around the world. Thematically, I was able to identify the following requirements: research experience evidenced by journal publications and conference presentations, a referee report, a statement of purpose, funding, a test of English (International English Language Testing System - IELTS, Test of English as a Foreign Language - TOEFL, etc.), a research proposal and a first-class degree and/or MSc with a good grade.

First, I started with a research proposal. Research is all about solving problems while extending existing knowledge. Having studied marine science with a focus on marine microbiology and biotechnology at the master’s level and having read various publications on the problems associated with seafood, I decided to write a proposal on seafood-borne pathogens.

Second, I wrote and published journal articles from my MSc thesis, along with other personal write-ups. I also presented at conferences.

Third, I wrote a statement of purpose, encompassing my areas of interest, especially food safety and quality. The statement of purpose entails, why one wants to study at the doctoral level, why one chooses to solve the identified problem, skills acquired and the skills that still need to be acquired. These are the basis of a doctoral study.

Fourth, I approached my MSc lecturers and supervisor for a referee report. I requested a ‘To Whom It May Concern’ referee report. This enabled me to use the report for different applications without going back to my referees for a new report, which may be burdensome to them.

The fifth generic requirement was having a test of English. I searched for the best test I could perform. I decided to write IELTS (an academic module) with a target of 7.0 as an overall band while ensuring no band was less than 6.0 in any of the modules (writing, speaking, reading and listening). I obtained preparatory materials from those who had written the exam before and I also used the internet to prepare.

In addition to the above, I ensured my MSc grade was good enough (achieving a distinction) to improve my chances of obtaining a PhD admission. It took me almost a year to prepare and obtain the above requirements, on top of completing the MSc degree that I started in 2009.

## Lessons I learned

- Write a clear goal of where you are heading in your career.
- Do not be rigid with your timeframe.
- A delay in achieving your goal in a specified timeframe does not mean a denial of the goal.
- Identify the requirements to achieve your goal.
- Strategically plan how to meet the requirement.
- Identify a problem that you want to solve.
- Prepare ahead of time.
- Count the cost of studying overseas, especially if you do not have fixed funding for your tuition fees and living expenses.
- Learn from those ahead of you.

## The search

Having prepared the above-listed requirements, it was time to search for potential PhD supervisors. The right supervisor is important, as a supervisor is like a global positioning system that will guide you throughout the journey [1]. I sent out several emails to prospective supervisors in different parts of the world, including Australia, Malaysia, South Africa, Canada and New Zealand, to mention but a few. While some responded, the majority did not. Those that responded stated that they did not have the funding for research or were not able to accept new doctoral students. I was never discouraged by those negative responses. Rather, I persisted in my search for a supervisor.

I came across the University of Tasmania during one of my searches. I sent an email to one of the academics who is globally known in the field of food safety. He responded the same day, stating he did not have funding for my area of interest (seafood) but that he would ask his colleagues. His colleague was interested in my research proposal, but he preferred that I should work on meat spoilage. In the end, the admission was not successful, but I got an offer from an institute in the same university to work on seafood spoilage The offer came with both tuition fee scholarship and a living allowance stipend. This was a joyful moment for me, having waited for almost a year. I was able to acquire the same skills that I would have acquired if I worked on meat spoilage.

## Lessons I learned

- Not everyone will be interested in your research proposal.
- Determination, resilience and persistence are golden attributes for career progression.
- You can acquire the same skills through different pathways.
- While some applicants may secure supervisor and funding easily, it is not always the same for everyone; do not feel bitter or disappointed if you cannot secure a supervisor and funding immediately.

## The settling period

After receiving the offer, I embarked on preparing the documents for my family and myself to get a visa. I also needed to pay a health insurance fee, in order for one of the key documents that I required for the visa to be issued by the university. I sold my car to raise the money while a friend assisted with some money, too. We applied and got the visa. Moving a family of three with a 2-month-old baby was not easy. Changing the climatic environment was another challenge for us, aside from the fact that we had been used to the previous country of residence (Malaysia) having stayed there for over 5 years. My primary supervisor was very supportive of our settling down. He ensured everything went well for us and even provided some clothing and household items as we came during Spring (a cold and windy season in Tasmania!).

The environment was very strange and adapting to such cold conditions was very challenging. No sooner than that, our baby developed a skin infection due to the harsh weather. Combining my nascent study and family became another challenge. We do not have a car for easy mobility and that means I have to abandon some academic tasks to run errands for the family. It took us time to be able to settle down and acculturate [2,3]. Now, 5 years on, we have acclimatized and enjoy living in Tasmania. While it has been a tough journey, it was greatly helped by the support we received from my primary supervisor and my ensuring I took time to ensure the work-life balance was correct.

## Lessons I learned

- Work-life balance is important for a successful career.
- Settling down in a foreign country or a new job is a gradual process.
- You need a good support system for you to settle down easily.

## The scientific period

As often said, the learning curve of a PhD student is not a straight-line but rather a curve [4]. Starting from the changing of my research topic due to the interest in the seafood industry, I went through a closure of a company due to toxin contamination of the seafood as a result of algal bloom, learning various statistical packages, managing huge dataset, failed experiments, shortage of funds, breakdown of equipment, the crashing of my laptop while analyzing my data, long waits for laboratory consumables and loneliness in the laboratory for many hours. My experience was not an exception. However, all these have enabled me to acquire and sharpen skills such as critical-thinking, problem-solving, time and project management, communication to diverse audiences and conflict management and have improved my expertise in my chosen field of research. The latter end of my first year and the whole of my second year was the best period for learning, productivity and acquiring new skills. This was because my proposal was already settled, I had passed the qualifying exam often called confirmation of candidature, ‘CoC’, among PhD students and my research direction was already known.

## Lessons I learned

- The learning curve of PhD, has never been a straight line. There are inevitable ups and downs.
- The roughness of the journey makes learning holistic by shaping all areas of life and prepare for a future career.
- There are unexpected and unavoidable situations that you may come across.

## The sad & joyful moments

My first year went well as planned aside from the changing of my research focus. While my second year was filled with the above challenges, my third year started with the crashing of my laptop while analyzing one of my huge dataset. All efforts to fix the machine proved abortive, but I had at least backed up my dataset. Without the backup, I would have lost a whole year of my study. Second, that would have extended my study unnecessarily. Sadly, while trying to navigate through my scientific journey, I lost two of my siblings to the cold hands of death. I became depressed and lost interest in the whole journey, knowing full well that I would not set my eyes on them again even after acquiring the doctorate degree. The intimacy that had existed between us and the constant phone calls even though we had not physically seen each other for years made the grieving period worse. Often times, I still remember them and weep.

However, I completed all my experiments, analyzed those huge dataset, wrote and submitted my thesis for examination. The joyful moment came when I received an email that my thesis passed examination and that the Dean of Graduate Research had approved my graduation with a doctorate degree. My joy knew no bounds.

## Lessons I learned

- There are unexpected situations that you may come across. Some could be avoided or prevented, some are bound to happen and are meant to better shape you.
- Determination and resilience are vital for successful completion of a doctoral degree.
- Mental depression among PhD students is real and could be product of various factors such as lost family members and failed experiments, among other things.
- You need the support of others such as your family members, friends, supervisor (s) and the university itself to successfully complete the doctoral journey.
- When things are rough, always remember why you started the journey in the first instance.
- Ensure you have the right motive before embarking on a doctoral journey because of the inherent challenges that are ahead.

## Conclusion

Despite all the challenges that I encountered during my doctoral research journey, I was able to complete the degree within 4 years as stipulated by the university. This was because of me having the right motives, a good support system, determination and resilience. You should always remember that you cannot tread the journey alone. While ruminating on the whole journey, I can confidently say that it was worth acquiring a doctoral degree because you become better informed, transformed, inspired and prepared for the future. The skills acquired during the journey will last into any future job and prepare you for any career pathway.
